# Evolutionary insights and functional diversity of gasdermin family proteins and homologs in microorganisms

**DOI:** 10.3389/fimmu.2024.1371611

**Published:** 2024-03-20

**Authors:** Shule Wang, Tingbo Ma, Xiaoyi Xia, Leiliang Zhang

**Affiliations:** ^1^ Department of Clinical Laboratory Medicine, The First Affiliated Hospital of Shandong First Medical University & Shandong Provincial Qianfoshan Hospital, Jinan, Shandong, China; ^2^ School of Clinical Medicine, Shandong Second Medical University, Weifang, Shandong, China; ^3^ Department of Pathogen Biology, School of Clinical and Basic Medical Sciences, Shandong First Medical University & Shandong Academy of Medical Sciences, Jinan, Shandong, China

**Keywords:** gasdermin, microorganisms, programmed cell death, immune response, evolutionary parallels

## Abstract

The gasdermin protein family and its homologs in microorganisms have gained significant attention due to their roles in programmed cell death, immune defense, and microbial infection. This review summarizes the current research status of gasdermin proteins, their structural features, and functional roles in fungi, bacteria, and viruses. The review presents evolutionary parallels between mammalian and microbial defense systems, highlighting the conserved role of gasdermin proteins in regulating cell death processes and immunity. Additionally, the structural and functional characteristics of gasdermin homologs in microorganisms are summarized, shedding light on their potential as targets for therapeutic interventions. Future research directions in this field are also discussed to provide a roadmap for further investigation.

## 1 Introduction

Microorganisms not only possess immune systems but also play a significant role in biological evolution, particularly in the realm of immunity due to their pathogenic characteristics (1–3). The conflict between capsid-encoding organisms (such as viruses) and ribosome-encoding organisms (such as fungi and bacteria) has been a major driving force behind microbiological evolution within the framework of natural selection (4). As a result, a wide range of diverse antivirus defense systems have evolved in all cellular life forms, accompanied by counter-defense systems in many viruses. All the living organisms including bacteria and fungi constantly face a barrage of diverse viruses, triggering the evolution of multiple defense systems (5–7).

New research has revealed intriguing evolutionary similarities in defense-related proteins between multicellular animals (metazoans) and microbes, shedding light on the increasing number of shared components in immune systems across different kingdoms. One of the most fascinating proteins is the gasdermin (GSDM) family. At least 50 bacterial GSDMs (bGSDMs) have been identified, forming a distinct clade from their eukaryotic homologs and are present throughout bacterial and archaeal genomes (8–10). Although genomic analysis has detected the presence of gasdermin in archaea (9, 10), there is a lack of functional experimental studies on gasdermin in archaea. GSDMs in mammals possess a C-terminal repressor domain, cytotoxic N-terminal domain, and flexible linker domain (11). The activation of GSDMs involves the proteolytic removal of the inhibitory C-terminal domain, a mechanism that has been confirmed through structural analysis of related genes and proteins, as well as the recent discovery of GSDMs in viruses (12). GSDM family proteins play a critical role in a highly inflammatory immune cell death program called pyroptosis (13). In mammals, pyroptosis is initiated by inflammasomes, which detect cytosolic contamination or perturbation, resulting in the activation of caspase-1 or caspase-11/4/5 (14). These caspases cleave GSDM to generate the GSDM-NT domain, which forms large pores in the plasma membrane, leading to swelling and rupture (15). The release of specific cytokines through these pores includes the proinflammatory cytokines interleukin (IL)-1β and IL-18, which exert toxic effects on the surrounding cells and tissues (14). Recent studies have uncovered the presence of GSDM-containing immune systems in microorganisms such as *Bradyrhizobium tropiciagri*, *Vitiosangium* sp., *Runella*, *Podospora anserina*, *Neurospora crassa*, and poxviruses (16–19). GSDMs from microorganisms exhibit structural similarities to those found in host cells, but they may induce different biological behaviors. Analyzing the gene sequences and domains of GSDMs reveals conserved amino acid sequences and specific domains, providing a basis for further investigation into their function and mechanism of action.

Heterokaryon incompatibility (HI) in fungi and abortive infection (Abi) in bacteria are defense mechanisms involving regulated cell death to prevent the spread of mycoviruses in fungi and bacteriophages in bacteria (20, 21). GSDM homologs, namely fungal GSDM (fGSDM) and bGSDM, play a crucial role in controlling these defense-related cell suicide strategies (20, 21). The discovery of the molecular regulators of GSDM cytotoxic effects in fungi and bacteria provides valuable insights into the evolutionary links between these organisms and mammalian pyroptotic pathways.

However, viruses possess the ability to continually evolve and acquire host genes to manipulate host biology, facilitating their replication (22). A recent study demonstrated that a GSDM homolog present in poxvirus can interfere with inflammatory caspases, suppressing inflammasome signaling and pyroptosis (19). This finding highlights the dual role of GSDM homologs in immunological processes across different species and their involvement in viral infections. This review focuses on exploring the participation of GSDM homologs in the immune systems of bacteria and fungi, as well as the specific roles played by viral GSDM homologs in host cells, providing a detailed understanding of their mechanisms.

## 2 GSDM homologs in fungi

**Figure 1:**
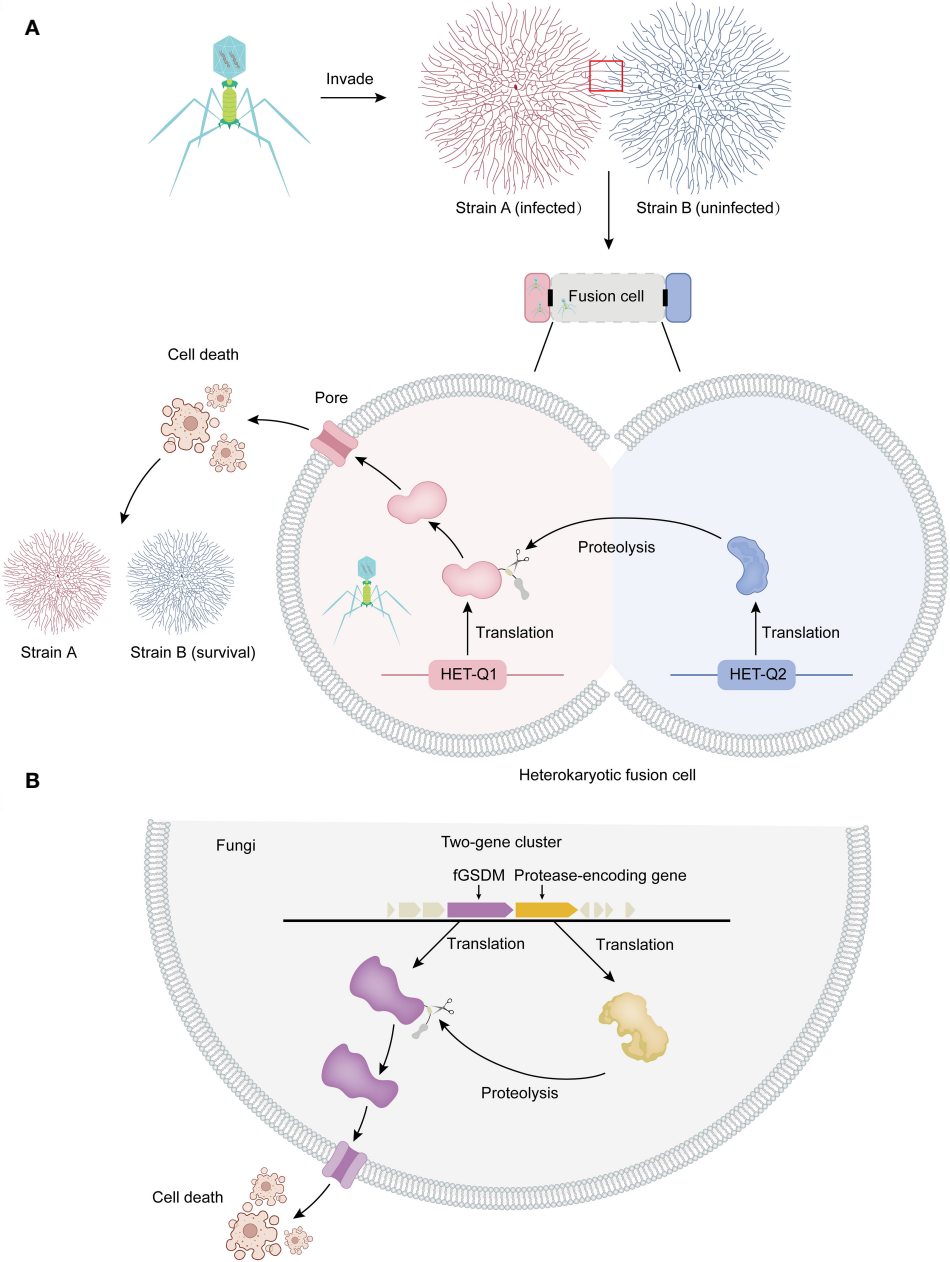
Allorecognition and programmed cell death in fungi. **(A)** Allorecognition mechanism: Allorecognition occurs when genetically distinct fungal individuals come into contact. The fusion of hyphae from genetically incompatible isolates leads to the formation of heterokaryotic cells. Within these fusion cells, the HET-Q2 subtilase enzyme activates the fGSDM protein called HET-Q1 through proteolysis. Once activated, fGSDM forms pores on the plasma membrane, specifically triggering programmed cell death in the heterokaryotic cells. This defense mechanism ensures the separation of genetically different strains, preventing the transmission of mycoviruses and other harmful replicons from one strain to another. Thus, it safeguards the integrity of strain B. **(B)** Proteolysis and programmed cell death: In fungi, genes encoding fGSDM and proteases are often clustered together. The expressed proteases facilitate the proteolysis of fGSDM, leading to programmed cell death.

The discovery and study of fGSDMs are within the context of programmed cell death induced by allorecognition, which is the ability of an organism to distinguish between self and nonself individuals of the same species (23, 24). This programmed cell death occurs when genetically distinct individuals of the same fungal species fuse hyphae. This fusion triggers regulated cellular death in newly formed heterokaryotic cells, preventing cytoplasmic mixing and maintaining separation between incompatible colonies (25, 26). The rejection of nonself, known as heterokaryon (or vegetative) incompatibility (HI or VI) (27), plays a crucial role in this process. This allorecognition-mediated programmed cell death serves as a defense mechanism against genome exploitation and ensures that viruses do not spread between genetically distinct strains of the fungus (**Figure 1**) (24). The genes responsible for allorecognition, known as *het* (*heterokaryon*) or *vic* (*vegetative incompatibility*), are bi- or multiallelic and highly polymorphic. Allorecognition systems often consist of two tightly linked genes (25). Genetic analyses of vegetative incompatibility in filamentous fungi, such as *Neurospora crassa*, *Podospora anserina*, *Aspergillus nidulans*, and the plant pathogen *Cryphonectria parasitica*, have shown that incompatibility is genetically controlled by multiple, unlinked het or vcg (vegetative compatibility group) loci (28). GSDM homologs are abundant and widely distributed in the fungal kingdom, with approximately 1900 identified members encoded in the genomes of 400 different species, particularly within the Ascomycota phylum. On average, each genome contains 5 gasdermin genes, with more than 12 species possessing over 10 fGSDM, and some fungi even encoding more than 20 gasdermin homologs in their genomes (17, 18).

### 2.1 Regulator of cell death-1 (RCD-1)

*Neurospora* is the genus of a group of filamentous fungi, but the term is commonly used as a nickname for the most extensively studied species, *Neurospora crassa*, which has been a model eukaryotic organism for almost a century. *Neurospora* grows at a rapid rate and, importantly, has uncovered several remarkable and unexpected genetic mechanisms that act to combat invasive DNA (29). One of these mechanisms involves RCD-1-dependent cell death. Approximately 180 fungal species were found to have at least one rcd-1 homolog, with an average of five and a median of three homologs per sequenced genome. Several *Trichoderma* species have a high number of *rcd-1* homologs, with some strains carrying up to 23 genes in their genome. This shows that the *rcd-1* gene family, including the allorecognition determinant in *Neurospora crassa*, is widespread among Ascomycota fungi (18). The NCU05712 locus was identified as a RCD-1, which controls programmed cell death in germinating asexual spores of *Neurospora crassa*. The *rcd-1* alleles in *Neurospora crassa* populations are highly polymorphic and exhibit signs of balancing selection. Genomic rearrangements in the *rcd-1* locus may have played a role in the evolution of this allorecognition system in Neurospora, and such rearrangements could be a common mechanism in fungal incompatibility systems. At the *rcd-1* locus, two incompatible alleles, *rcd-1-1* and *rcd-1-2*, were identified, and the coexpression of these alleles triggered vacuolization and cell death. Interestingly, an *rcd-1-2* allele was found inserted into an *rcd-1-1* allele, disrupting the gene structure. This observation highlights the frequent occurrence of genomic rearrangements in non-self recognition loci (18).

Germling-regulated death (GRD), a type of cell death that occurs in germinating spores, has been molecularly characterized in filamentous fungi. It involves an NLR (nucleotide-binding domain leucine-rich repeat) protein that controls an allorecognition and programmed cell death process specifically at the germling stage, which refers to germinated asexual spores (30). Notably, *rcd-1* is responsible for regulating GRD. It is worth mentioning that most *het* genes do not regulate cell death in this context, but rather in mature hyphae. The molecular basis of rcd-1-dependent cell death has been explored. Through *in silico* analyses, RCD-1 is revealed as a distant homolog of the N-terminal pore-forming domain of GSDM. RCD-1 localizes to the cell periphery, and its cellular localization is associated with conserved positively charged residues on predicted amphipathic α-helices, similar to murine GSDMD. Like GSDM, RCD-1 binds to negatively charged phospholipids such as cardiolipin and phosphatidylserine, and interacts with liposomes containing these lipids. Furthermore, the RCD-1 fGSDM variant has been found to bind to cardiolipin. These results suggest that mitochondria capable of synthesizing cardiolipin could be a target for some fGSDMs (31). This hypothesis is supported by the fact that human GSDME and GSDMD were observed to permeate mitochondria. However, unlike mammalian GSDMs, the cytotoxic activity of fungal RCD-1 GSDM-like proteins results from the interaction of antagonistic RCD-1 variants (17). Additionally, oligomers of RCD-1 were associated with the cell death reaction, further supporting the evolutionary relationship between GSDM and RCD-1 (24). It was also found that the antagonistic RCD-1-1 and RCD-1-2 proteins interact exclusively during the cell death reaction in mammalian cells, and that RCD-1 forms large supramolecular assemblies in *Neurospora crassa* (24). Further experiments are needed to elucidate the exact mechanism by which RCD-1 regulates cell death.

### 2.2 HET-Q1 and HET-Q2

Several *het* genes, including *het-Q1* and *het-Q2*, have been identified and shown to induce cell death when coexpressed with incompatible alleles of the same gene (allelic HI systems) or with incompatible alleles of a different gene (nonallelic HI systems). The recognition of non-self is often mediated by protein-protein interactions that exhibit a temperature-sensitive characteristic (32).

The *het-Q1* gene encodes one fGSDM consisting of 278 amino acid residues, while the *het-Q2* gene encodes a 388 amino-acid-long serine protease from the S8 family (17). The cytotoxic activity of HET-Q1 is controlled by proteolysis. During the cell death reaction in the presence of a subtilisin-like serine protease called HET-Q2, HET-Q1 undergoes the loss of a approximately 5 kDa C-terminal fragment (**Figure 1A**). Mutational analyses and successful reconstitution of the cell death reaction in a heterologous host (*Saccharomyces cerevisiae*) suggest that HET-Q2 cleaves HET-Q1 at the P1 amino acid residue F238, resulting in the removal of 40 residues from the C-terminal end of the fGSDM, thereby inducing cell death during the allorecognition cell death reaction in *Podospora anserine* (**Figure 1A**) (17).

### 2.3 Q2-L proteases control the fGSDMs in a variety of fungal species

Analysis of the genomic landscape of *het-Q1* homologs in fungi has revealed that the vast majority of *GSDM* genes are clustered with protease-encoding genes. Approximately 80% of *GSDM* genes are located in close proximity (+/- 10 kb region) or adjacent to protease-coding genes in fungi, forming two-gene clusters (**Figure 1B**) (17). These protease-coding genes are referred to as *Q2-L* (similar to *het-Q2*). In approximately 60% of cases, Q2-L contains a serine-S8 protease that exhibits characteristics similar to those of a subtilase, akin to HET-Q2. Notably, approximately 17% of Q2-L proteins contain the CHAT (caspase HetF associated with tetracyclic peptide repeats [TPRs]) protease domain instead of subtilase. Both types of proteases (subtilase-like and caspase-related) regulate GSDM-dependent cell death in fungi, likely in the context of organismal defense (17). Several of the most abundant HET-Q2–like protein architectures feature annotated domains comprising tandem-repeat motifs such as TPR, ANK (ankyrin), or WD40 repeats (17). The TPR repeat is 34 amino acids long and consists of two α-helices forming an α-helix-turn-α-helix motif (33). The ANK repeat is a 33 amino acid motif, with the structure of a single motif starting with a β-turn followed by two antiparallel α-helices and ending with a loop that connects to the next repeat (34).

## 3 GSDM homolog in bacteria

**Figure 2:**
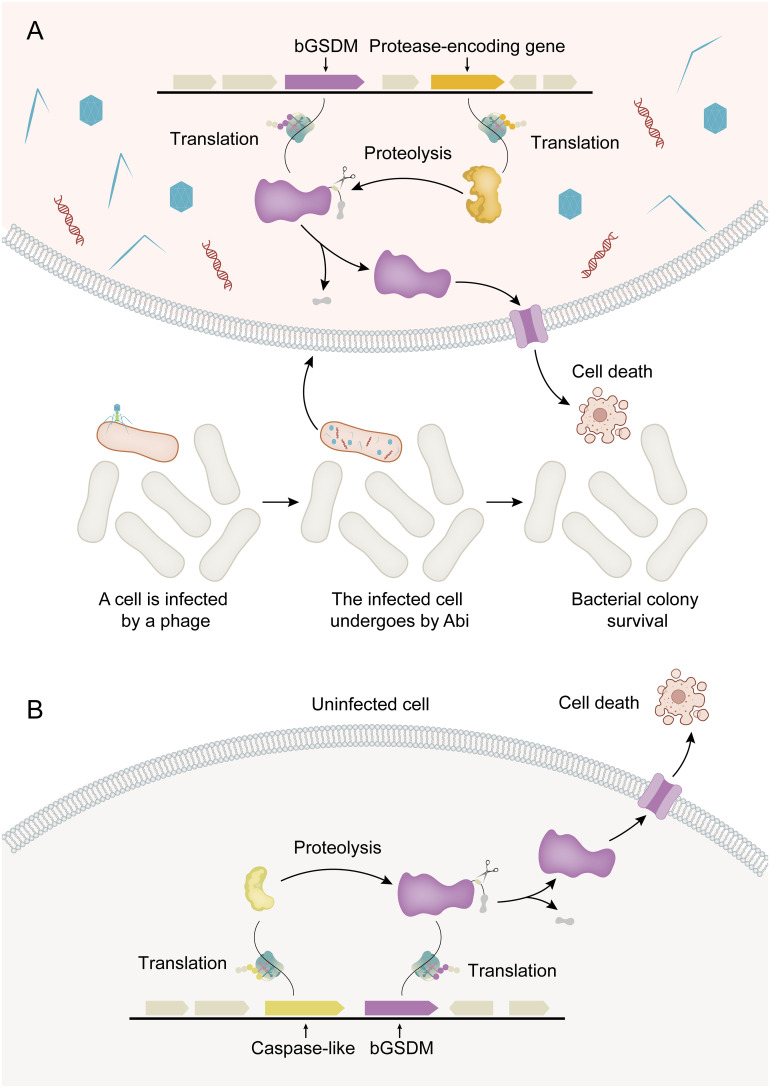
Activation mechanisms for bGSDM cytotoxicity. **(A)** Abi (abortive infection) is a defense-related cell suicide strategy in bacteria. In phage-infected bacteria (shown in red), bGSDM is activated by proteolysis, resulting in programmed cell death that ultimately leads to the survival of the bacterial colony. **(B)** In the *Runella* system, *Runella* bGSDM is also subjected to proteolysis by proteases, resulting in programmed cell death even in the absence of phage infection.

The discovery of bGSDMs initially came from the analysis of gene content in bacterial defense islands, which are large genomic regions where defense-associated genes and putative Abi systems cluster together (35). Abi is not a defense system itself but rather an immune strategy observed in various defense systems encoded by bacteria. The core principle of this strategy is that when a bacterial cell senses infection, it undergoes programmed cell death before the phage can complete its replication cycle. This prevents the release of mature phage particles, thereby halting the spread of the phage epidemic to nearby cells and ensuring the survival of the colony (5). It has been discovered that bGSDMs can induce bacterial cell death through Abi systems, thereby ensuring the survival of the bacterial colony (**Figure 2A**). In summary, bacteria encode bGSDMs that are activated by dedicated proteases, providing defense against phages and inducing cell death. The presence of bGSDM-encoding genes in the operon is crucial for the antiphage defense response, highlighting the important role of bGSDMs in mediating Abi (16).

Structural studies of bGSDMs have revealed a conserved pore-forming domain that remains inactive due to a buried lipid modification (16). In other words, the last few residues were buried in another part of bGSDM to maintain the inactive state of the entire bGSDM. Truncation of the C-terminal peptide in the *Runella*, *Bradyrhizobium*, or *Vitiosangium* bGSDM constructs resulted in arrested cell growth, confirming that the C-terminal peptide is necessary to maintain bGSDM autoinhibition. In mammals, the GSDM-N domain is functionally significant, leading to membrane binding and lysis induction, and is shared among the GSDM family. The GSDM-N domains of GSDMD, GSDMA3, and GSDMA are capable of binding to membrane lipids, such as phosphoinositides and cardiolipin, resulting in membrane-disrupting cytotoxicity in mammalian cells (36). This effect also has been observed in *E. coli* containing recombinant expression of a cholesterol-targeting pore-forming toxin from *Clostridium perfringens*, as well as in artificial liposomes made of major bacterial membrane lipids like cardiolipin and phosphatidylethanolamine (36). Additionally, bGSDM exhibits a high structural similarity to the twisted β-sheet cores of the pore-forming domains of GSDMD and GSDMA3. The crystal structures of bGSDMs from *Bradyrhizobium tropiciagri* and *Vitiosangium* sp. reveal a shared overall architecture that resembles the mammalian GSDM N-terminal domain (NTD), including a twisted central anti-parallel β-sheet and conserved connecting helices and strands throughout the periphery (16). These findings suggest that bGSDMs may operate through a similar functional mechanism. Regarding the C-terminal domain (CTD), the structures of bGSDM have shown that they lack the large α-helical CTD present in mammalian GSDMs, which is crucial for maintaining the autoinhibited state of these proteins. Surprisingly, despite the short length of C-terminal peptide, the structures of bGSDM adopt a conformation similar to the inactive complex of mammalian GSDMs (16). The short C-terminal peptide from bGSDM stabilizes the inactivated state of bGSDM and exerts an inhibitory function. For example, in the case of *Bradyrhizobium* bGSDM, the residues F245 and F247 are positioned along the surface equivalent to the mammalian β9 strand and the α1 helix, with further support from contacts between N21 and the peptide backbone. A *Bradyrhizobium*-specific β-strand extending from N21 to L24 extends off the equivalent of the β9 strand and is reinforced by a short parallel β-strand from F253 to D255. The residue F253 of *Bradyrhizobium* bGSDM interacts with the palmitoylated residue C3, and similar hydrophobic contacts have been observed in the structures of bGSDMs from *Runella* and *Vitiosangium*. It should be noted that protein palmitoylation, a type of protein lipidation involving the post-translational addition of a lipid to a protein, specifically entails the thioesterification of a sixteen-carbon saturated fatty acid (palmitate) to an internal cysteine residue. This process can influence protein localization, accumulation, secretion, stability, and function by altering membrane affinity (37). The C-terminal peptide of *Bradyrhizobium* bGSDM terminates below the equivalent of the β2 strand and is supported by hydrogen bonds from R27 to the L256 backbone and N29 to E258 (16). This suggests that bGSDM has evolved alternative mechanisms to maintain its inactive state and regulate its activity. The stabilization of the core structure in bGSDM and the inhibitory function of its peptide chains are likely responsible for preventing the uncontrolled activation of the protein. In bGSDM systems, the inhibitory CTD is wrapped up in a stable hydrophobic domain. Hydrolysis of the CTD by proteases can induce the activation of bGSDM. These findings provide valuable insights into the structural and functional diversity of GSDMs across different organisms. Further studies are needed to elucidate the specific mechanisms by which bGSDM achieves inhibition and how it differs from the regulation of mammalian GSDMs. Furthermore, with the exception of DFNB59, all members of the GSDM family in mammals are conserved and consist of highly conserved N-terminal and C-terminal fragments that are separated by a central flexible and viable linker containing pore-forming activity (38). However, it remains an open question as to how common the inhibition mechanisms are among all bGSDMs and/or fGSDMs. Such knowledge can contribute to a better understanding of GSDM biology and potentially guide the development of therapeutic interventions targeting GSDM-related pathways.

The cytotoxicity of bGSDMs can vary depending on the induction method in heterologous systems. For instance, the expression of certain bGSDM-protease systems in *E. coli* can result in potent cellular toxicity even without phage infection (**Figure 2B**). Strong toxicity has been observed in a *Runella* system, which requires bGSDM palmitoylation (16). Similarly, the cytotoxic activity of GSDMD in mammals relies on its ability to associate with and integrate into cellular membranes, leading to oligomerization and the formation of pores that disrupt membrane integrity (39). Moreover, the cytotoxic activities of bGSDM proteins vary significantly in different situations. For instance, the *Runella* bGSDM forms a ring-like pore with a width of approximately 50 Å and an inner diameter ranging from 200 to 300 Å. The pores formed by *Runella* bGSDM within liposomes measured around 240 to 330 Å. Additionally, some smaller pores measuring 130 to 190 Å in diameter were observed from the reconstituted cleavage of a bGSDM from a metagenomic Bacteroidetes scaffold (16). Each bGSDM protein displays a unique range of pore diameters, indicating that the variations in GSDM pore size are a predetermined feature regulated by protein sequence specificity. In combination, the size range of bGSDM pores extends well beyond that of characterized human GSDMs, and the architecture of the pores is a diverse characteristic across the evolution of GSDM proteins (40).

## 4 GSDM homologs in viruses

In addition to eukaryotic and prokaryotic cells, GSDM homologs have also been found in viruses. A recent study identified vaccinia virus (VACV) A47 as a homolog of GSDMs through structural modeling, which was further confirmed by an X-ray crystal structure of A47 (19). The authors conducted a structural homology screen for VACV, eliminating most nonspecific results and yielding 60 well-characterized homologs of host proteins, including A47. In this study, AlphaFold2 (41) was utilized to demonstrate that eptesipox GSDM shows significant structural homology with various metazoan GSDM CTDs and the predicted AlphaFold2 model of VACV A47 (19). However, eptesipox GSDM possesses an additional α-helix off its N terminus that is not present in metazoan GSDMs (19).

Furthermore, the authors performed complementary searches for viral GSDMs in poxvirus proteomes. These searches identified poxvirus GSDMs in vespertilion-, lepori-, and avipoxviruses, as well as ortho- and centapoxviruses (19). Some poxvirus genomes within the mammal-infecting orthopox and centapox clades contain two copies of viral GSDM: one “long” form and another somewhat truncated “short” form. Together with avipox GSDM, these forms cluster into three distinct clades (19). While the GSDMs in these viruses are diverse, they phylogenetically cluster in comparison to vertebrate GSDM regulatory domains, indicating a single acquisition event (19).

In a previous study, it was demonstrated that A47 contains numerous CD8+ T cell epitopes in VACV, but it is not essential for viral growth (42). The recent study found that the expression of VACV GSDM rescued cells from LPS-induced cell death, similar to the effect observed with the expression of the regulatory domain of GSDMD (19). This suggested that A47 might enhance infectivity by reducing host mortality. By investigating the GSDM-mediated cell death induced by A47 in the absence of caspase activity, researchers found that A47 did not inhibit GSDMD NTD-mediated killing in any system (19). Therefore, it is proposed that A47 interferes with the ability of active inflammatory caspases to process their substrates (19). Based on the homology between A47 and GSDMD, it is suggested that A47 may competitively inhibit the processing of GSDMD by active inflammatory cysteases. In future research, it will be necessary to determine whether A47 acts as a competitive inhibitor that blocks the GSDMD exosite of inflammatory caspases or functions as a depressant to promote caspase degradation. Additionally, the presence of GSDM homologs in other viruses should be investigated further.

## 5 Summary and prospect

The GSDM family proteins are a widely distributed class of proteins found in diverse organisms, serving various biological functions, including innate immunity, hair cell maintenance and induction of auditory pathway neuron activity in humans. This review provides an overview of the recent research progress on GSDMs and their homologs in microorganisms, aiming to provide valuable insights for related research. The presence of GSDM homologs in microorganisms suggests that the GSDM family may be a shared component of the innate immune systems of certain mammals and microorganisms. These findings have emerged in the context of immune systems that are resistant to bacteriophages in bacteria and fungi, with related studies reporting evolutionary similarities between the antiviral defense systems of fungi, bacteria, and mammals. For example, RCD-1 and HET-Q1, which are fGSDM proteins, have been identified respectively in the genomes of *Neurospora crassa (* 18, 24) and *Podospora anserina* (17, 26), two model Ascomycete species. These discoveries shed light on the molecular mechanisms of programmed cell death occurring during non-self recognition in germinating conidia of *Neurospora crassa* and *Podospora anserina*. On the other hand, bGSDMs were discovered by analyzing the gene content of bacterial defense islands (16). Both bGSDM and fGSDM function by regulating programmed cell death. Microbial GSDMs, especially bGSDM, can adopt various circular pore structures to achieve pyroptosis, similar to mammalian GSDMs. While the fungal clade of microbial GSDMs lacks high-resolution structural information, future research efforts should focus on obtaining high-resolution structural data on the cytotoxic pores of fGSDM.

These discoveries of GSDM and its homologs in fungi and bacteria suggest a potential common origin of pyroptotic-like cell death in microorganisms and pyroptosis in animals. Furthermore, recent studies have shown that GSDM homologs are also present in viruses (19). However, it is important to note that the gene for the GSDM homolog in viruses was captured from the host gene, as exemplified by the identification of the GSDM homolog A47 in poxviruses (19). The VACV A47 protein interferes with the ability of inflammatory caspases to process their substrates following inflammasome activation in order to execute its function. Further studies are needed to establish the specific mechanisms by which A47 does so and to shed light on how different viral GSDMs work to benefit viral infection.

Despite some progress in the study of GSDMs and their homologs in microorganisms, there are still many questions that need to be explored. Future research should delve into the structure-function relationship of GSDM and its homologs, reveal the specific molecular mechanisms of GSDM-induced cell pyroptosis, explore the role of GSDM and its homologs in different diseases and pathological processes, and study the interaction between GSDMs and other biomolecules, as well as their synergistic effects in biological processes. Further investigation of these questions will provide a more comprehensive understanding of the biological significance and potential applications of GSDMs and their homologs.

## Author contributions

SW: Software, Writing – original draft. TM: Software, Writing – original draft. XX: Software, Writing – review & editing. LZ: Conceptualization, Writing – review & editing.
